# Hémangiome hépatique géant de découverte tomodensitométrique

**DOI:** 10.11604/pamj.2016.25.241.10363

**Published:** 2016-12-20

**Authors:** Traore Abdoulaye Ababacar, Alaoui Lamrani Youssef

**Affiliations:** 1 Service de Radiologie du CHU Hassan II, Fès, Maroc

**Keywords:** Hémangiome hépatique géant, tomodensitométrie, douleurs abdominales, Giant hepatic hemangioma, computed tomography, abdominal pain

## Image en médecine

Nous rapportons l’observation d’une patiente de 48 ans, qui présente depuis 1 et ½ une augmentation progressive du volume abdominal, des douleurs abdominales intermittentes sans troubles du transit, associées à une altération de l’état général. L'examen clinique trouve une volumineuse masse abdomino-pelvienne mobile et dure à la palpation, non douloureuse. Le bilan biologique initial notait une anémie à 9,6 g/dL hypochrome microcytaire, sans saignement extériorisé, une cholestase anictérique avec des phosphatases alcalines à 151 UI/L et la Gamma GT à 71 UI/L. Le reste du bilan était normal. Ce tableau clinico-biologique a été complété par une échographie abdominale qui a montré une volumineuse lésion d’échostructure hypoéchogène hétérogène mal délimitée, présentant un contact intime avec le foie, sans vascularisation spécifique au doppler. Le complément TDM abdomino-pelvien montre une volumineuse masse au dépend du foie droit étendue en bas dans la région pelvienne mesurant approximativement 48x30x27cm dans les différents plans. Cette masse était hypodense en contraste spontané et présentait un rehaussement en motte après contraste, attestant d’une lésion angiomateuse. Par ailleurs, la masse englobait le tronc porte et des branches veineuses intra hépatiques gauches. Les autres branches des veines sus-hépatiques et la VCI étaient laminées. Le diagnostic d'hémangiome géant remanié, avec des zones de nécrose et de thrombose a été retenu. La patiente a été mise sous antalgiques, vu l’absence d’une issue thérapeutique.

**Figure 1:**
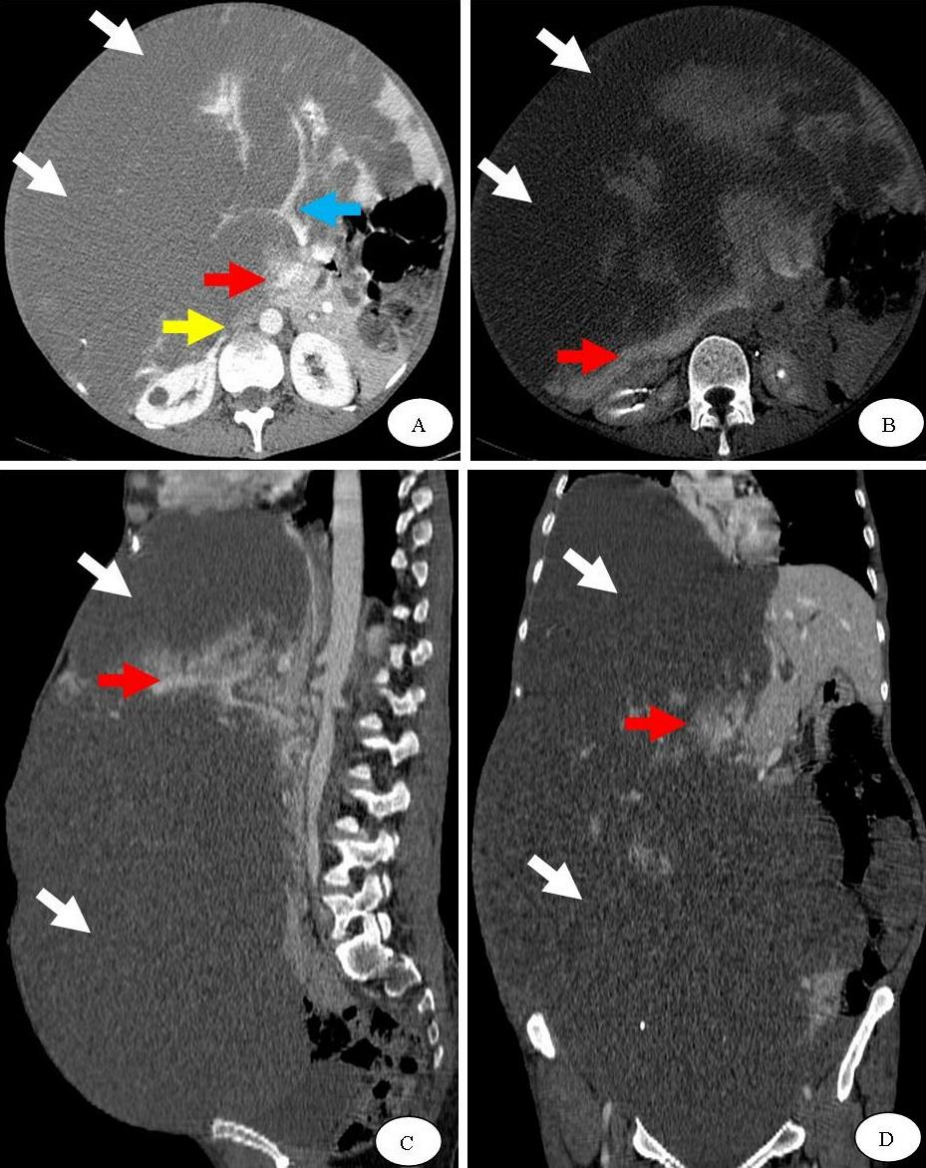
Coupes TDM axiale après contraste (A), au temps tardif (B) et après reconstructions sagittale (C) et coronale (D) montrant une volumineuse masse du foie droit étendue en bas dans la région pelvienne (flèche blanche). Cette masse est massivement liquéfiée et présente par endroit un rehaussement en motte après contraste (flèche rouge), orientant vers un angiome géant hépatique. Par ailleurs, noter un aspect grêle mais perméable du tronc porte (flèche bleue). La VCI est laminée (flèche jaune)

